# Evaluation of Product Yield and Fatty Acid Composition of Three Flax (*Linum usititassimum* L.) Varieties Depending on Different Sowing Dates

**DOI:** 10.3390/life15030483

**Published:** 2025-03-17

**Authors:** Nazlı Aybar Yalınkılıç, Şilan Çiçek Bayram, Sema Başbağ

**Affiliations:** 1 Department of Plant Production and Technologies, Faculty of Applied Sciences, Muş Alparslan University, Mus 49000, Türkiye; 2 Institute of Science, Dicle University, Diyarbakır 21100, Türkiye; silan.cicek@tarimorman.gov.tr; 3 Department of Field Crops, Faculty of Agriculture, Dicle University, Diyarbakir 21100, Türkiye; sbasbag@dicle.edu.tr

**Keywords:** yield components, ecological factors, alphalinolenic acid (ALA), flaxseed, biplot analysis, correlation analysis

## Abstract

The flax (*Linum usititassimum* L.) plant can grow in various agroecological environments. However, there are some factors that affect the production and quality of flax. Sowing date is one of the important factors determining crop productivity. In this study, four different sowing dates (20–22 March, 1–3 April, 15–18 April and 30 April, respectively) were applied to determine the appropriate sowing date in terms of yield and oil quality in three flax varieties (Sarı Dane, Kara Kız, Beyaz Gelin). The study results showed that by delaying sowing, the seed filling period of the plant was exposed to high temperatures, and this caused decreases in seed yield. In other words, the most positive results in terms of agronomic characteristics were obtained from the first sowing date. In this respect, the highest oil yield of 760 kg ha^−1^ and the highest oil content of 34% were obtained from the Sarı Dane variety. Similarly, flaxseed showed high values in terms of alphalinolenic acid (54.25%), palmitic acid (6.36%), stearic acid (7%), oleic acid (22.54%) and linoleic acid (14%) at the first and second planting times, while these rates decreased relatively as the planting time was delayed. According to the results of the two-year study, it was determined that the ecological conditions of the region were suitable for flax cultivation. However, it is clear that delaying the sowing date causes significant decreases in both the agronomic traits of flax and the oil quality for industrial purposes. In this respect, considering the ecology of the region, sowing flax in the last week of March or the first week of April is suitable for optimum yield and oil quality.

## 1. Introduction

Vegetable oils account for approximately 79% of annual global oil production [1,2]. Flax is one of the oldest cultivated oilseed crops, and its seeds are used directly or by processing in many commercial areas [3]. Flaxseed is rich in alphalinolenic fatty acid (ALA), omega-3, secoisolariciresinol diglucoside (SDG), fiber and lignin. Due to the bioactivity of the content of these products, they support the health of the body and are used in daily consumption [4,5]. It is known that flaxseed contains more ω-3 polyunsaturated fatty acids than soybeans, fish, corn and marine algae [6,7]. The fatty acids contained in flax seeds have many benefits for human health. It has been found to protect against cancer, atherosclerosis, hormonal problems, neurological problems and coronary heart disease [8,9]. In addition, interest in flaxseed has increased due to the potential to obtain products containing higher levels of essential and beneficial oils by feeding them to farm animals. Flax, when included in animal rations in various forms (whole seed, meal, oil supplements or as hulls) contributes to the increase in the nutritional value of the feed. Moreover, oil obtained from flaxseed is also used for biodiesel production [10,11]. The fact that biofuel production from oilseeds can be an environmentally friendly alternative to diesel fuels increases the importance of this issue. For these reasons, interest in flaxseed has increased in recent years [7]. With the increasing interest in flaxseed, strategies need to be determined to increase product productivity in order to meet the increasing demand [12].

Flax has an important position in the world economy due to its wide range of industrial uses. However, unprecedented climate change could also negatively impact flax production [13]. Therefore, implementing appropriate agricultural strategies to increase the productivity and oil quality of the flax plant will contribute to balancing the effects of climate change. Flax can be grown in a variety of ecological environments. In addition, it has a high ability to adapt to unfavorable environmental conditions and can adapt to soils with a low nitrogen content [14]. The aim of flax cultivation is to obtain high levels of products as well as quality products from the unit area. Sowing date is one of the most important factors affecting the yield and yield characteristics of the products [15]. Determining the optimum sowing date for a seed is one of the important environmental factors affecting the germination and growth stages. Therefore, predicting the appropriate sowing date for plants during the growing season both positively affects plant development and allows farmers to benefit from climatic factors such as temperature and precipitation [16].

Early sowing of flax plants may negatively affect germination due to low temperatures. It may also cause diseases and pests to appear early. Conversely, delaying sowing may prevent the plant from reaching physiological maturity. Therefore, determining the best sowing date for flax is an important method to reduce the effects of biotic and abiotic stress factors [14,17]. Sowing date is one of the important and effective factors that influence flax seed yield and oil quality [18]. Many authors have reported that the highest yield was obtained from early sowing [19,20,21]. Sowing date should be determined according to the ecological factors of each region. In this respect, it is of great importance to correctly determine the sowing date of oil crops according to the region where they are grown. In addition, determining the appropriate sowing date in arid climate regions is necessary to increase potential crop productivity and use available irrigation water effectively [22].

The effect of sowing dates on flax yield and fatty acid composition is complex and may vary depending on climatic conditions, cultural practices and genotype. Therefore, further research is needed to improve planting practices to increase flax yield and nutrient composition. Determining the appropriate sowing date for a particular region or environment and selecting the most productive varieties will help optimize flax productivity. To date, no comprehensive evaluation has been made on flax genotypes or optimum sowing dates for summer annual flax production in Muş Province located in eastern Türkiye. Therefore, this study was designed to determine the crop productivity and oil quality of three flax cultivars at four different sowing dates.

## 2. Materials and Methods

### 2.1. Design and Maintenance of the Trial Area

In this study, Sarı dane, Kara kız and Beyaz Gelin flax varieties were used as the material. This study was conducted in a randomized block factorial design with three replications. Thus, the first factor was the flaxseed variety and the second factor was the three different sowing dates. The varieties used in this experiment and their sowing dates are given in Table 1.

In the study, the field was plowing before the seeds were planted. Each plot consisted of 6 rows, 4 m long and 20 cm apart. Flax seeds were planted manually at a depth of 3 cm with approximately 2 cm spacing [14]. In addition, the distance between plots was determined as 1 m, and the distance between replicates was determined as 4 m. No diseases or pests were observed in the experimental area during the growing season. Weed control was carried out manually when necessary. Therefore, the research was conducted under ecological conditions, and no chemical fertilizer or chemical spraying was applied. In addition, the experimental area was not irrigated, and the study was conducted under rainfall-based conditions. Harvesting was carried out manually when the capsules were completely yellow. During harvest, two rows were left as edge effects, and the following observations were taken from the remaining four rows in the middle: plant height (cm), number of branches per plant, number of capsules per plant, number of seeds per capsule, capsule diameter (mm) and capsule length (mm). Twenty mature plants were randomly selected from each plot to determine these characteristics.

### 2.2. Site Climate Conditions Description

This research during the flax growing seasons from 2021 to 2022 as a two-year field study was conducted in Muş Province, located in the eastern region of Türkiye. Muş Province has a continental climate with significant temperature differences throughout the year and with an average annual temperature of 11.5 °C and an average rainfall of 689 mm. Muş Province is located between 38.29°–39.29° northern latitudes and 41.06°–41.47° eastern longitudes (altitude 1350 m). Before sowing, soil samples (0–30 cm) were collected from the trial site and chemical analyses were performed. The soil analysis results of the trial area are given in Table 2. Accordingly, the soil structure was clayey–loamy, the soil pH was 7.70 and the organic matter content was medium. The soil pH was measured with distilled deionized water and 1 M KCl at soil solution ratios of 1:1, 1:2.5 and 1:5.

The monthly average air temperature during the flax growing season was similar to the average long-term yearly temperature (Table 3). The average temperature in 2021 was higher in March, April and May compared to 2022 and the long-term averages. The highest temperatures were seen in July and August. According to 2021, 2022 and the long-term averages, the highest precipitation occurred in March. In 2021 and 2022, the precipitation in March was greater than the long-term average. Flax plants grow well in cool spring periods. This shows that the climate conditions of the region are suitable for flax cultivation. Therefore, the optimum sowing date for flax would be mid-March and early April, when average temperatures reach 8–10 °C. In addition, when the flax growing season of the region was examined, it was seen that the highest rainfall occurs in March (Table 3). There was also some rainfall in April and May.

### 2.3. Nutritional Composition Profile of Seeds

Immediately after harvest, the yield per plot was weighed, and samples were taken from each plot to determine the oil content (the Soxhlet method; SIST EN ISO 659:1998). All analyses were performed in three replicates for each sample. To determine the dry matter (DM) content, all plant samples were oven-dried at 65 °C to constant weight. Samples were ground to pass through a 1 mm screen and made ready for analysis. Approximately 10 g of sample was transferred to a glass bottle equipped with a Soxhlet apparatus. The oils taken into the organic solvent (150 mL of hexane) were evaporated under vacuum at approximately 40 °C, and then the remaining oil was dried in the oven at 105 °C for 35 min. Then, the oils were weighed on a precision scale and calculated as a percentage (%). Thus, the oil content of the seeds was determined [23]. The nitrogen content of the flaxseeds was determined using a Thermo Elemental Analyzer. For nitrogen determination, 2–3 mg of flaxseed was placed in special tin capsules and placed in the automatic sampling chamber of the device (Thermo Scientifc’s Flash 2000/MAS 200R, Waltham, MA, USA) The oven temperature of the device was set to 950 °C, and the detector temperature was set 65 °C. Helium was used as the carrier gas, and the flow rate was set to 100 mL per minute. A linear calibration was obtained for N using sulphanilamide as a reference standard material. Thus, N determination was carried out by a thermal conductivity detector. The protein content was obtained by multiplying the amount of nitrogen by 5.85 [24]. The total carbohydrate content was calculated according to oilseed application in the NIRMASTER (BÜCHI, Flawil, Switzerland) instrument operating with the near infrared spectroscopy method (NIR).

### 2.4. Oil Yield (kg ha^−1^)

The oil yield was calculated according to the following formula [25]:

Oil yield (kg ha^−1^) = Oil content (%) × Seed yield (kg ha^−1^)

### 2.5. Fatty Acid Profile of Seeds

The fatty acid composition of the oil samples was determined according to the standard for the determination of fatty acid methyl esters (FAMEs) by gas chromatography at Dicle University Science and Technology Application and Research Center. Accordingly, 0.1 g of flaxseed oil was taken into 15 mL centrifuge tubes and 10 mL of hexane solvent was added. Then, 0.5 mL of KOH was added to the tubes. Samples taken from the upper phase of the solution were placed in a Shimadzu AOC-20i automatic injector. A GCMS-TQ8030 (Shimadzu, Kyoto, Japan) instrument with a Flame Ionization Detector (FID) was used for essential fatty acid analysis. Helium was used as the carrier gas. The FID temperature was 250 °C, pressure was 250 kPa and split ratio was 1/100. After waiting for 5 min at 140 °C, the column temperature increased by 4 °C per minute to reach 240 °C, and after waiting for 15 min, it was increased to 250 °C. Thus, the results obtained after injecting 1 µL of each sample into the gas chromatography device were compared with the GC-FID chromatogram obtained during a total of 50 min of analysis of the “Supelco Fame mix 37” standard mixture. As a result of the analysis, the amounts of palmitic acid (C16:0), stearic acid (C18:0), oleic acid (C18:1), linoleic acid (C18:2) and alphalinolenic acid (ALA-C18:3) contained in the flaxseed oil were given as percentages [14,26].

### 2.6. Statistical Analysis

The data obtained for all traits were subjected to analysis of variance (ANOVA). The SAS statistical package program version 9.1 was used to analyze the data obtained as a result of the study, and the differences between the means were compared according to the LSD_0.05_ test. Bartlett’s homogeneity test was performed before combining the years. Thus, after determining that the years were homogeneous, combined analysis was performed. In addition, some agronomic data obtained from the study were analyzed with the GT biplot (Genstat version 14) method reported by [27]. Yield–trait interactions were calculated based on the average of years.

## 3. Results and Discussion

### 3.1. Agronomic Traits

The combined analysis of variance (ANOVA) results of the examined traits are given in Table 4. Accordingly, the variety, sowing time and variety × sowing date interaction showed significant effects on all studied agronomic traits. However, the year, year × sowing date interaction, year × variety interaction and year × variety × sowing date interaction were found to be not statistically significant for all agronomic traits.

The effects of the different sowing dates on flax varieties are given in Table 5. Accordingly, the sowing date x variety study showed that a delay in the sowing date caused significant reductions in the plant height of the three varieties. The highest plant height was obtained from the Kara Kız (55 cm) variety at the first sowing date, followed by the Beyaz Gelin (51 cm) and Sarı Dane (48 cm) varieties, respectively. It is assumed that the decrease in plant height resulting from delayed sowing may have been due to the shorter vegetation period. Our results are in agreement with those obtained by Sankari Choi et al., who found that differences in sowing date caused a wide variation in mean plant height among flaxseed cultivars [28,29]. Additionally other researchers have also reported that plant height decreases with the delay in sowing date [14,15,30]. Similarly, as the sowing date was delayed, the number of branches per plant decreased significantly.

The highest number of branches at the first sowing date was obtained from the Kara Kız (17 per plant), Beyaz Gelin (14 per plant) and Sarı Dane (11 per plant) varieties, respectively. The lowest number of branches was taken from the Sarı Dane (four per plant) variety at the fourth sowing date, followed by the Beyaz Gelin (seven per plant) and Kara Kız (eight per plant) varieties. These results are consistent with previous studies, which reported significant decreases in the number of branches with a delayed sowing date [17,18,31,32]. In the three flax varieties, there was a significant decrease in the number of capsules as the sowing date was delayed (Table 4). The highest number of capsules was obtained from the Kara Kız variety at the first sowing date. The number of capsules of the Kara Kız variety varied between 58 and 23 per plant. The variety with the most variable number of capsules depending on sowing date was Beyaz Gelin. Accordingly, the number of capsules of this variety was 38 per plant at the first sowing date, while it was 22 per plant at the fourth sowing date. Drej and Noaman, reported that the number of capsules per plant varied between 64.18 and 39.18 depending on different sowing dates, and a significant decrease in the number of capsules occurred with delaying sowing [31]. Maurya et al. confirmed that an early sowing date for flax gave the best results in terms of number of capsules per plant [20]. Ganvit et al. reported that the sowing date has a significant effect on the number of capsules and the number of seeds per capsule [21]. Our findings in the present study are consistent with the results of many researchers reporting that a significant decrease in the number of capsules occurs with delayed sowing [33,34,35].

Depending on the sowing date for the number of seeds in the capsule, the Sarı Dane variety had values between 10-5, the Beyaz Gelin variety had between 9-6 and the Kara Kız had variety between 11-6 per capsule. As the sowing date was delayed, the number of seeds in the capsules decreased significantly. The highest number of seeds per capsule was observed at the first sowing date in all three varieties. On the contrary, the lowest values for the number of seeds in the capsule were obtained at the fourth sowing date. This may be due to the negative effect of temperature on pollination and ovary viability due to delayed sowing dates. This is because by delaying sowing, the seed filling period is exposed to high temperatures and this negatively affects seed formation. Sohair et al. observed that the number of capsules per plant was significantly affected by sowing dates [36]. Vidyalaya explained that an earlier sowing date gives better results for the number of seeds per capsule and seed yield [37]. The results of the present study are in agreement with previous results reporting that the number of seeds in the capsule decreased significantly with delaying sowing [20,21,34,35]. Similarly, the capsule length and capsule diameter decreased with a delayed sowing date.

High oil yield is one of the most important goals of cultivating flax. Significant differences were determined in the sowing date x variety interaction in terms of oil yield (Table 5). Depending on four different sowing dates, the oil yield varied between 310–707 kg ha^−1^. This critical decrease in oil yield with the delay in the sowing date showed that the sowing date has a significant effect on oil yield. Thus, the highest oil yield was obtained from the Sarı Dane variety at the first sowing date. However, the lowest oil yield was obtained from the Kara Kız variety at the fourth sowing date. Delaying the sowing date causes physiological stages such as seed filling and seed fixation to take place within ecological periods with higher temperatures. This leads to a decrease in efficiency. Mirzaie et al. reported that the oil yield of flax plant in Iran varied between 77.59–1112 kg ha^−1^ depending on the sowing date, and unfavorable last growing season conditions such as intense heat significantly reduced the oil yield on the third and fourth sowing dates [14]. Gallardo et al. stated that the oil yield in Argentina varied between 45 kg ha^−1^ and 644 kg ha^−1^ and that delaying sowing negatively affected the oil yield [38]. In studies carried out under controlled conditions, it was determined that exposure of seeds to high temperatures (>30 °C) during the maturation phase reduced the number of seeds per capsule and also reduced the oil yield and quality [39]. Generally, the yield of a plant is directly affected by the growth period and environmental conditions. Thus, if the plant’s growth period is sufficient and the ecological conditions are suitable, the yield will be higher. Our results are consistent with researchers who argue that the oil yield decreases as the sowing date in flax is delayed [11,38,40]. On the contrary, Zheljazkov stated that the sowing date x variety interaction was not significant in terms of oil yield in flaxseed [41].

### 3.2. Climatic Conditions

Temperature and precipitation play an important role in determining the sowing date of flax. Rainfall after sowing flax seeds is an important factor for seed germination and plant growth. Rainfall in March and April meets the early water needs of the plant and does not require farmers to irrigate additionally. Rainfall requirements during the vegetative and flowering periods are very important for the yield parameters of flax. Flaxseed is sensitive to drought during early seed development and flowering periods. Therefore, it should be remembered that the seed yield and oil content can be maximized by maintaining adequate soil moisture during these periods [42]. Jarošová et al. found that flax grown in years characterized by low rainfall resulted in significantly lower seed yields as well as lower thousand-seed yields compared to years with normal or increased rainfall levels [43]. The adverse effects of water scarcity in the early stages of flaxseed development have been reported by many researchers [44,45]. Casa et al. reported that flaxseed yield in Italy varies greatly with climatic conditions, with temperature and water scarcity being the most likely factors to affect yield [46]. Weather conditions have a significant impact not only on the oil yield but also on the quality and fatty acid composition of linseed oil [33,44]. Additionally, the heat and drought experienced in midsummer allow the plant to reach harvest maturity. However, Luhs and Friedt found that high temperatures during the seed-filling period negatively affected the seed yield, accelerating maturation and reducing the oil content [47]. As a result, when the climate conditions were evaluated, it was determined that the region was suitable for flaxseed cultivation. Additionally, considering the climatic conditions of the region, it has been determined that the most suitable sowing date for flax is the last week of March or the first week of April.

### 3.3. Biplot Analysis

**Figure 1:**
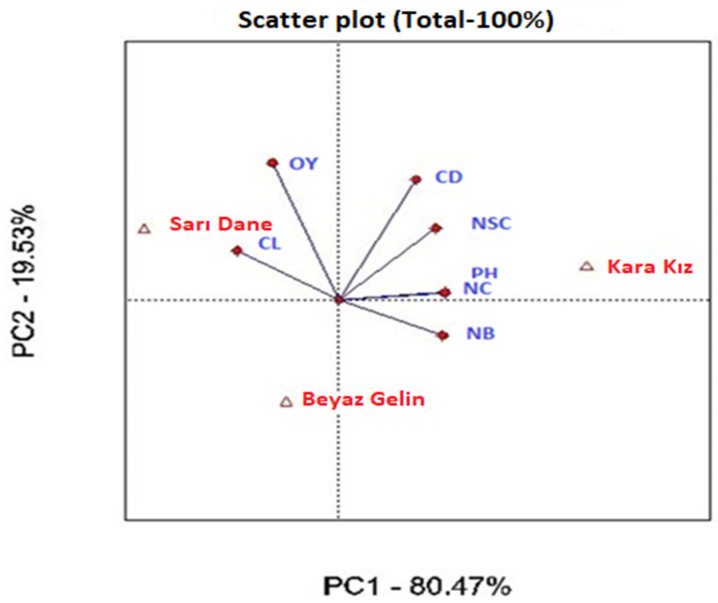
Variety–trait relationship based on biplot. CL: capsule length (mm), CD: capsule diameter (mm), NSC: number of seeds per capsule (per capsule), NC: number of capsules (per plant), PH: plant height (cm), NB: number of branches (per plant), OY: oil yield (kg ha^−1^), PC: principal components.

**Figure 2:**
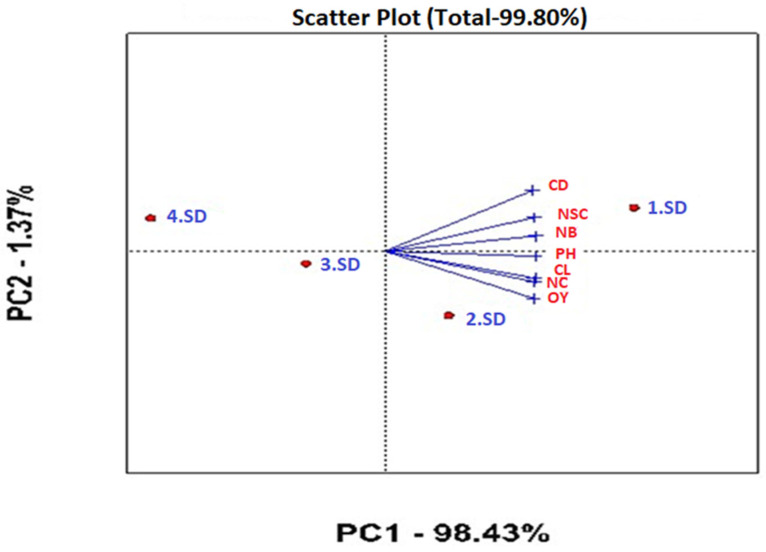
Sowing date–trait relationship based on biplot. CL: capsule length (mm), CD: capsule diameter (mm), NSC: number of seeds per capsule (per capsule), NC: number of capsules (per plant), PH: plant height (cm), NB: number of branches (per plant), OY: oil yield (kg ha^−1^), PC: principal components. 1. SD: first sowing date, 2. SD: second sowing date, 3. SD: third Sowing date, 4. SD: fourth sowing date.

Data on the investigated traits of genotypes at different sowing dates were analyzed with the biplot method (Genstat version 14), as suggested by [27]. Data on agronomic traits were determined according to the sowing date and average of the varieties. The angle between the vectors in the biplot chart examines the relationship between the examined traits. In this context, the widening of the angle between two features reduces the positive relationship, while the narrowing of the angle indicates a strong positive relationship between the two features. Biplot analysis is a two-dimensional graphical representation of genotype, trait and genotype × trait interaction, consisting of the first two principal components (PC1-PC2). Figure 1 was constructed based on the performance of the varieties for each trait. Principal component analysis is appropriate when a small number of components explain a significant portion of the total variance (i.e., the first two or five components explain more than 60% of the total variation) or when components are selected with eigenvalues greater than one. In this context, PC1 represented 80.47% of the variation, while PC2 represented 19.53%, with both axes explaining a total of 100% of the variation (Figure 1). Varieties positioned near certain traits represent good results according to the parameters on which they are positioned. For example, while the Sarı Dane variety had high values for the OY and CL traits, the Kara Kız variety showed superior properties in terms of the CD, NSC, PH and NC traits. The Beyaz Gelin variety showed the weakest performance in terms of the examined traits. Similarly, Figure 2 was created based on the performance of the examined traits at different sowing dates. PC1 represented 80.47% of the variation, while PC2 represented 19.53%, with both axes explaining a total of 100% of the variation. In the biplot model examining the sowing date–trait relationship, PC1 and PC2 represented 1.37% and 98.43% of the variation, respectively, explaining a total of 99.80% of the variation (Figure 2). It was determined that all the features examined in the vector presentation of the biplot chart gave the best results on the first and second sowing dates. On the contrary, a decrease in the performance of all traits examined was observed in the third and fourth sowing times.

### 3.4. Nutritional Composition and Fatty Acid Profile

The changes in the nutrient composition of the three flax varieties at the different sowing date are given in Table 6. The results of mean comparisons showed that the delay in sowing date caused a decrease in nutrient composition. The highest oil, protein and carbohydrate compositions were obtained from the Sarı Dane variety at the first sowing date. The oil content of the Sarı Dane variety varied between 34% and 19%, the carbohydrate content varied between 8% and 6% and the protein content varied between 24% and 18%. In addition, the oil content of the Beyaz Gelin variety was between 31% and 17%, the protein content was between 23% and 18% and the carbohydrate content was between 7% and 6%. Similarly, the oil, protein and carbohydrate contents of the Kara Kız variety were in the range of 16–30%, 17–23% and 6–7%, respectively (Table 6).

The oil content of flaxseed is reported to be approximately 40% [19]. However, some studies have shown that the oil content in different agro-climatic zones and in seeds of different varieties may vary between 23.28% and 46% [20,23]. Unfavorable climatic conditions such as high temperature on the third and fourth sowing dates significantly reduced the oil, protein and carbohydrate contents. In the present study, the changes in oil, protein and carbohydrate content can be attributed to the effects of environmental factors such as high temperature and water scarcity during the seed filling period on these characteristics. Although the nutritional components of oilseeds are genetically controlled, they are also known to be affected by genotype, geographical location, growing season and agricultural practices [15,48]. Shaikh et al. reported that the oil content in flaxseeds varied between 40% and 40%, and the highest oil content was obtained from early sowing [49]. The decrease in the nutritional composition of flaxseeds with a delay in sowing has also been supported by other researchers [50,51,52].

Table 7 shows the fatty acid contents of the oil obtained from the three flaxseed varieties according to the different sowing dates. The most attractive feature of flax is its significant content of alphalinolenic acid, which is the end product of three desaturation steps. According to the study results, the sowing date had a significant effect on the fatty acid composition of the flax seeds. The oleic and alphalinolenic acid contents decreased significantly with the delay in the sowing date. The highest values for alphalinolenic acid fatty acid were obtained from the Beyaz Gelin (54%), Sarı Dane (53%) and Kara Kız (51%) varieties at the first sowing date, respectively. The results of the current study showed a decrease in the C18:3 acid percentage as the sowing date was delayed. These results are consistent with findings reporting that high temperature during seed development causes a decrease in linoleic and alphalinolenic acid [17,52,53].

The oleic fatty acid content of the three flax varieties varied between 20.00–20.37% (Sarı Dane), 20.15–20.34% (Beyaz Gelin) and 20.98–22.54% (Kara Kız). The results obtained from the study showed that the amount of C18:3 fatty acid was 45.37–54.67% [14] and 42.40–51.07% [50]. Xie et al. examined the fatty acid profile of flax and determined the highest values of palmitic acid, stearic acid, oleic acid, linoleic acid and alphalinolenic acids as 5.43%, 5.72%, 27.31%, 14.61% and 48.59%, respectively [25]. Gallardo et al. found that the palmitic acid content of flax seeds varied between 6.55–6.57%, stearic acid between 7.33–7.44%, oleic acid between 28.95–31.43%, linoleic acid between 15.23–15.82% and alphalinolenic acid between 47.24–48.68% on two different sowing dates (June 11 and July 30, respectively) [38]. Pali and Mehta reported that the alphalinolenic acid content varied from 33.14% to 54.82%, while the average values of linoleic, oleic, stearic and palmitic acids were 15.88%, 27.76%, 6.26% and 6.07%, respectively [54].

The alphalinolenic acid content, known to be high in flaxseed, is generally over 50% [48,55,56]. Hatanaka et al. reported in their study in a temperate region of Japan that sowing dates had a significant effect on the fatty acid composition of flax seeds [57]. In addition, they stated that the highest yield and quality was obtained from the first sowing time and that the probable reason for this situation was that the seed maturation was exposed to high temperatures as planting was delayed. It has been confirmed by many researchers that the sowing date has a significant effect on the oil composition of flax [19,25,58]. It is assumed that the changes in the oil composition of flax varieties at different sowing dates may be due to climatic factors. So, high temperatures experienced during the seed filling period due to late sowing practices affected the fatty acid composition. This shows that the fatty acid composition of flax is affected by genotype, cultivation techniques, ecological conditions and sowing time.

### 3.5. Correlation Analysis

Correlation coefficients of some agronomic traits for the different sowing dates are given in Table 8. A significant and high correlation was found between plant height and branch number. This shows that plant height significantly affects the branch number. There was a significant and positive correlation between the number of branches and number of capsule traits at all four sowing dates. The effect of the number of branches on the oil yield was found to be significant in the first and second sowing dates. In addition, the number of capsules per plant had a significant effect on the oil yield at all sowing dates. This shows that the number of branches, the number of capsules and the number of seeds per capsule are directly important in terms of the oil yield. Mirshekari et al. reported that there was a significant and positive correlation coefficient among the yield components of flax, and the correlation between the number of capsules and the number of seeds per capsule was especially significant [17].

## 4. Conclusions

In this study, significant statistical differences were detected both among the varieties and among the examined traits by delaying the sowing date. According to the study results, the delay in sowing negatively affected both the yield and the quality of the oil. Although significant variability was observed in the flaxseed depending on the genotype and sowing date, it showed important agronomic characteristics that made it suitable for introduction in the region where the study was conducted. When the climatic conditions of the region and the results obtained from the study were evaluated together, it was determined that the most suitable sowing date for flax was the last week of March and the first week of April. Moreover, by delaying sowing, the seed filling period of the plant was exposed to high temperatures, and this caused a decrease in seed yield. Although the best results were obtained at the first sowing date in this study, the results obtained at the third and fourth sowing times also showed that flax could be an alternative product for farmers. Flaxseed can be preferred by farmers because of its high adaptability, low input cost in agriculture and its oil’s various uses. In addition, the short vegetation period of flax and the fact that it meets most of the water it needs during its growing period through rainfall make the plant an indispensable product for marginal agricultural areas.

## Figures and Tables

**Table 1:** Sowing and harvesting dates of the three flax varieties examined in 2021 and 2022.

Varieties	Year	Sowing Dates	Harvest Dates
Sarı dane, Beyaz Gelin, Kara kız	2021	20 March	16 June
		1 April	25 June
		15 April	10 July
		30 April	22 July
	2022	22 March	17 June
		3 April	22 June
		18 April	14 July
		30 April	25 July

**Table 2:** The soils analysis results of 2021–2022 experiment areas.

Soil Depth (cm)	Soil Structure	Electrical Conductivity (dSm^−1^)	pH	Total Nitrogen (%)	Organic Matter (%)	Assumable Phosphorus (mg kg^−1^)	Exchangeable Potassium (mg kg^−1^)	Magnesium (mg kg^−1^)
0–30	Clayey–loamy	0.61	7.70	0.15	2.21	14.3	552	0.58

**Table 3:** Monthly temperature and precipitation distribution for the two years of the experiment and in the multi-year average (30 years) of Muş province.

	Average Temperature (°C)			Precipitation (mm)			Humidity (%)		
Months	2021	2022	Multi-Year (30 Years)	2021	2022	Multi-Year (30 Years)	2021	2022	Multi-Year (30 Years)
January	−5.5	−6.2	−5.3	94.0	88.0	103.9	85.0	83.9	82.0
February	−0.3	−2.6	−2.7	49.8	44.8	67.6	80.8	86.3	79.7
March	3.9	−0.5	3.2	166.4	203.4	131.3	69.8	83.6	71.4
April	13.6	11.7	11.2	7.8	28.0	67.1	48.8	52.3	55.3
May	18.7	13.8	15.8	11.6	80.0	72.2	39.9	60.4	54.1
June	23.4	22.0	21.5	0.6	19.0	25.8	26.7	41.3	39.4
July	27.2	26.1	26.2	0.4	1.0	9.6	27.9	27.1	27.7
August	26.3	27.2	26.5	10.4	0.0	3.5	28.9	24.8	25.7
Average	13.41	11.43	12.05	42.62	58.02	60.12	50.97	57.46	54.41

Muş Province Meteorology General Directorate.

**Table 4:** Combined analysis of variance of flax cultivars for agronomic traits.

SV	DF	PH	NB	NC	NSC	CL	CD	OC	OY
Y	1	51.802	45.321	422.345	0.123	0.172	1.243	1.753	1458
B(Y)	4	27.778	64.444	227.32	12.027	0.014	0.509	5.874	2527
SD	3	146.816 **	662.833 **	988.25 **	95.83 *	31.815 **	5.271 **	2.055 **	4788 **
YxSD	3	114.885 ^ns^	30.429 ^ns^	83.567 ^ns^	0.265 ^ns^	0.245 ^ns^	0.281 ^ns^	1.542 ^ns^	2022 ^ns^
V	2	244.111 **	293.777 **	124.562 **	2.216 **	5.404 **	5.443 **	81.09 **	671.13 **
YxxV	2	23.282 ^ns^	22.625 ^ns^	98.281 ^ns^	5.105 ^ns^	0.621 ^ns^	0.328 ^ns^	3.506 ^ns^	156.80 ^ns^
SDxV	6	323.374 **	200.000 **	266.832 **	2.6387 **	1.944 **	0.323 **	15.98 **	290.581 *
YxSDxV	6	21.661 ^ns^	32.582 ^ns^	15.282	2.4218 ^ns^	7.015 ^ns^	1.529 ^ns^	1.051 ^ns^	254.800 ^ns^
Error	44	23.172	18.367	18.22	22.015	6.710	0.056	3.420	2135
CV (%)		2.873	7.521	4.623	6.540	1.759	2.934	3.675	2.696

SV: sources of variation, DF: degrees of freedom, Y: year, B: block, V: variety, CV: coefficient of variation, PH: plant height (cm), NB: number of branches (per plant), NC: number of capsule (per plant), NSC: number of seeds (per capsule), CL: capsule length (mm), CD: capsule diameter (mm), OC: oil content (%), OY: oil yield (kg ha^−1^), ^ns^, *, **: non-significant and significant at the 5% and 1% probability levels, respectively.

**Table 5:** Sowing date x variety interaction of the investigated agronomic traits (2021, 2022).

Plant Height (cm)									
	Sarı Dane			Beyaz Gelin			Kara Kız		
	2021	2022	Ave.	2021	2022	Ave.	2021	2022	Ave.
1. SD	47 ± 0.6	49 ± 0.4	48 ± 0.5 ^A^	49 ± 0.1	52 ± 0.3	51 ± 0.2 ^A^	56 ± 0.5	53 ± 0.1	55 ± 0.3 ^A^
2. SD	43 ± 0.4	45 ± 0.2	44 ± 0.3 ^AB^	46 ± 0.3	47 ± 0.1	47 ± 0.1 ^AB^	47 ± 0.6	50 ± 0.4	49 ± 0.5 ^B^
3. SD	40 ± 0.6	42 ± 0.4	41 ± 0.5 ^B^	40 ± 0.2	44 ± 0.2	42 ± 0.2 ^B^	46 ± 0.4	44 ± 0.5	45 ± 0.4 ^B^
4. SD	37 ± 0.5	39 ± 0.2	38 ± 0.3 ^B^	36 ± 0.6	39 ± 0.2	38 ± 0.4 ^BC^	40 ± 0.2	41 ± 0.2	41 ± 0.1 ^BC^
Average	42	44		43	46		48	47	
LSD (_5%_)	1.055 **								
**Number of branches (per plant)**									
	**Sarı Dane**			**Beyaz Gelin**			**Kara Kız**		
	**2021**	**2022**	**Ave.**	**2021**	**2022**	**Ave.**	**2021**	**2022**	**Ave.**
1. SD	10 ± 0.06	12 ± 0.03	11 ± 0.04 ^A^	13 ± 0.03	15 ± 0.02	14 ± 0.02 ^A^	16 ± 0.03	18 ± 0.05	17 ± 0.04 ^A^
2. SD	7 ± 0.02	9 ± 0.01	8 ± 0.02 ^AB^	9 ± 0.02	11 ± 0.05	10 ± 0.03 ^AB^	14 ± 0.04	15 ± 0.02	15 ± 0.03 ^B^
3. SD	6 ± 0.01	8 ± 0.02	7 ± 0.01 ^AB^	8 ± 0.02	10 ± 0.09	9 ± 0.02 ^AB^	10 ± 0.02	12 ± 0.01	11 ± 0.02 ^BC^
4. SD	4 ± 0.05	5 ± 0.010	4 ± 0.07 ^C^	7 ± 0.08	8 ± 0.04	7 ± 0.01 ^B^	7 ± 0.02	9 ± 0.06	8 ± 0.04 ^C^
Average	7	9		9	11		12	14	
LSD (_5%_)	0.771 **								
**Number of capsule (per plant)**									
	**Sarı Dane**			**Beyaz Gelin**			**Kara Kız**		
	**2021**	**2022**	**Ave.**	**2021**	**2022**	**Ave.**	**2021**	**2022**	**Ave.**
1. SD	32 ± 0.3	34 ± 0.7	33 ± 0.5 ^A^	36 ± 0.8	40 ± 0.5	38 ± 0.7 ^A^	56 ± 0.4	60 ± 0.1	58 ± 0.2 ^A^
2. SD	29 ± 0.5	30 ± 0.10	30 ± 0.7 ^AB^	32 ± 0.4	34 ± 0.6	33 ± 0.5 ^AB^	44 ± 0.2	47 ± 0.10	46 ± 0.6 ^B^
3. SD	22 ± 0.4	28 ± 0.9	25 ± 0.7 ^B^	25 ± 0.3	29 ± 0.2	27 ± 0.3 ^B^	32 ± 0.4	36 ± 0.9	34 ± 0.5 ^C^
4. SD	15 ± 0.2	17 ± 0.6	16 ± 0.4 ^C^	20 ± 0.7	23 ± 0.4	22 ± 0.5 ^C^	21 ± 0.6	25 ± 0.5	23 ± 0.6 ^D^
Average	25	27		28	32		38	42	
LSD (_5%_)	0.285 **								
**Number of seeds capsule (per capsule)**									
	**Sarı Dane**			**Beyaz Gelin**			**Kara Kız**		
	**2021**	**2022**	**Ave.**	**2021**	**2022**	**Ave.**	**2021**	**2022**	**Ave.**
1. SD	9 ± 0.00	10 ± 0.04	10 ± 0.01 ^A^	8 ± 0.03	9 ± 0.06	9 ± 0.04 ^A^	10 ± 0.02	11 ± 0.04	11 ± 0.03 ^A^
2. SD	7 ± 0.05	8 ± 0.02	8 ± 0.03 ^B^	7 ± 0.04	8 ± 0.04	7 ± 0.04 ^AB^	8 ± 0.01	9 ± 0.02	9 ± 0.01 ^B^
3. SD	6 ± 0.03	7 ± 0.01	7 ± 0.02 ^B^	6 ± 0.01	7 ± 0.01	6 ± 0.00 ^AB^	7 ± 0.05	8 ± 0.04	7 ± 0.04 ^B^
4. SD	5 ± 0.02	6 ± 0.00	5 ± 0.01 ^AB^	6 ± 0.00	6 ± 0.06	6 ± 0.03 ^AB^	6 ± 0.03	7 ± 0.01	6 ± 0.02 ^BC^
Average	7	8		7	8		8	9	
LSD (_5%_)	0.109 **								
**Capsule length (mm)**									
**SD/V**	**Sarı Dane**			**Beyaz Gelin**			**Kara Kız**		
	**2021**	**2022**	**Ave.**	**2021**	**2022**	**Ave.**	**2021**	**2022**	**Ave.**
1. SD	8.98 ± 0.2	8.48 ± 0.3	8.73 ± 0.1 ^A^	8.52 ± 0.2	8.73 ± 0.5	8.62 ± 0.3 ^A^	8.13 ± 0.3	8.21 ± 0.1	8.17 ± 0.2 ^A^
2. SD	8.09 ± 0.2	8.16 ± 0.3	8.12 ± 0.2 ^A^	8.10 ± 0.1	8.13 ± 0.4	8.12 ± 0.2 ^A^	7.56 ± 0.2	7.69 ± 0.2	7.62 ± 0.2 ^B^
3. SD	7.65 ± 0.1	7.82 ± 0.2	7.73 ± 0.2 ^B^	7.18 ± 0.1	7.59 ± 0.4	7.38 ± 0.2 ^B^	7.01 ± 0.2	7.56 ± 0.2	7.28 ± 0.2 ^B^
4. SD	7.35 ± 0.1	7.45 ± 0.2	7.40 ± 0.1 ^B^	6.23 ± 0.2	6.55 ± 0.3	6.39 ± 0.4 ^BC^	6.21 ± 0.1	6.46 ± 0.3	6.33 ± 0.1 ^C^
Average	8	8		8	8		7	7	
LSD (_5%_)	0.771 *								
**Capsule diameter (mm)**									
	**Sarı Dane**			**Beyaz Gelin**			**Kara Kız**		
	**2021**	**2022**	**Ave.**	**2021**	**2022**	**Ave.**	**2021**	**2022**	**Ave.**
1. SD	7.23 ± 0.6	7.45 ± 0.7	7.74 ± 0.6 ^A^	7.02 ± 0.4	7.10 ± 0.7	7.06 ± 0.5 ^A^	7.78 ± 0.4	7.88 ± 0.7	7.83 ± 0.5 ^A^
2. SD	7.02 ± 0.3	7.10 ± 0.7	7.06 ± 0.5 ^AB^	6.72 ± 0.4	6.79 ± 0.8	6.75 ± 0.6 ^AB^	7.32 ± 0.6	7.40 ± 0.5	7.36 ± 0.5 ^AB^
3. SD	6.65 ± 0.5	6.88 ± 0.2	6.76 ± 0.3 ^B^	6.58 ± 0.6	6.63 ± 0.6	6.60 ± 0.6 ^AB^	7.22 ± 0.7	7.36 ± 0.5	7.29 ± 0.5 ^AB^
4. SD	6.38 ± 0.7	6.55 ± 0.6	6.46 ± 0.6 ^B^	6.49 ± 0.6	6.54 ± 0.6	6.51 ± 0.6 ^AB^	7.01 ± 0.7	7.08 ± 0.6	7.04 ± 0.6 ^B^
Average	6.82	6.99		6.70	6.76		7.33	7.43	
LSD (_5%_)	0.053 *								
**Oil yield (kg ha^−1^)**									
	**Sarı Dane**			**Beyaz Gelin**			**Kara Kız**		
	**2021**	**2022**	**Ave.**	**2021**	**2022**	**Ave.**	**2021**	**2022**	**Ave.**
1. SD	769 ± 15	752 ± 16	760 ± 15 ^A^	745 ± 17	730 ± 15	738 ± 16 ^A^	712 ± 13	702 ± 16	707 ± 15 ^A^
2. SD	668 ± 12	659 ± 9	664 ± 11 ^B^	640 ± 15	642 ± 15	641 ± 15 ^AB^	624 ± 12	627 ± 14	626 ± 14 ^B^
3. SD	543 ± 10	548 ± 9	546 ± 10 ^AB^	537 ± 14	532 ± 12	535 ± 13 ^B^	510 ± 11	516 ± 12	513 ± 13 ^C^
4. SD	430 ± 10	437 ± 8	434 ± 10 ^C^	429 ± 10	431 ± 10	430 ± 10 ^BC^	369 ± 10	371 ± 10	370 ± 13 ^CB^
Average	603	600		588	584		554	555	
LSD (_5%_)	25.768 **								

Values with different letters indicate significant groups at the 5% level. SD: sowing date, LSD: least significant difference. *, **: significant at the 5% and 1% probability levels, respectively.

**Table 6:** Nutrient composition (dry matter) of three flaxseed varieties according to four sowing dates (2021, 2022).

Oil Content (% of Dry Matter)									
	Sarı Dane			Beyaz Gelin			Kara Kız		
	2021	2022	Ave.	2021	2022	Ave.	2021	2022	Ave.
1. SD	32.26 ± 0.3	35.18 ± 0.4	34 ± 0.3 ^A^	30.23 ± 0.5	31.56 ± 0.4	31 ± 0.4 ^A^	29.28 ± 0.4	30.12 ± 0.3	30 ± 0.5 ^A^
2. SD	25.26 ± 0.1	27.39 ± 0.3	26 ± 0.2 ^B^	25.33 ± 0.4	27.32 ± 0.4	26 ± 0.3 ^AB^	23.77 ± 0.3	25.12 ± 0.0	24 ± 0.0 ^AB^
3. SD	20.18 ± 0.1	23.13 ± 0.1	22 ± 0.1 ^BC^	20.17 ± 0.4	20.39 ± 0.3	20 ± 0.2 ^B^	20.17 ± 0.0	20.72 ± 0.1	20 ± 0.4 ^AB^
4. SD	18.22 ± 0.1	19.32 ± 0.1	19 ± 0.1 ^C^	15.98 ± 0.3	18.22 ± 0.3	17 ± 0.3 ^B^	15.25 ± 0.1	16.45 ± 0.0	16 ± 0.1 ^B^
Average	24	26		23	24		22	23	
LSD (_%5_)	0.719 **								
**Protein Content (% of Dry Matter)**									
	**Sarı Dane**			**Beyaz Gelin**			**Kara kız**		
	**2021**	**2022**	**Ave.**	**2021**	**2022**	**Ave.**	**2021**	**2022**	**Ave.**
1. SD	23.56 ± 0.1	24.33 ± 0.3	24 ± 0.1 ^A^	22.18 ± 0.2	23.87 ± 0.4	23 ± 0.3 ^A^	22.37 ± 0.0	22.94 ± 0.1	23 ± 0.1 ^A^
2. SD	20.18 ± 0.2	21.16 ± 0.7	21 ± 0.5 ^AB^	20.03 ± 0.2	20.10 ± 0.3	21 ± 0.2 ^AB^	20.08 ± 0.1	21.16 ± 0.0	21 ± 0.1 ^AB^
3. SD	19.10 ± 0.0	19.25 ± 0.5	19 ± 0.2 ^AB^	18.45 ± 0.3	19.22 ± 0.3	19 ± 0.1 ^B^	18.27 ± 0.2	19.54 ± 0.2	19 ± 0.2 ^B^
4. SD	18.33 ± 0.1	18.92 ± 0.1	18 ± 0.1 ^AB^	17.56 ± 0.1	18.48 ± 0.2	18 ± 0.1 ^B^	16.54 ± 0.2	17.88 ± 0.2	17 ± 0.2 ^B^
Average	20	21		20	20		19	20	
LSD (_%5_)	0.393 **								
**Carbohydrate Content (% of Dry Matter)**									
	**Sarı Dane**			**Beyaz Gelin**			**Kara Kız**		
	**2021**	**2022**	**Ave.**	**2021**	**2022**	**Ave.**	**2021**	**2022**	**Ave.**
1. SD	7.87 ± 0.0	7.05 ± 0.2	8 ± 0.1 ^A^	6.58 ± 0.7	6.72 ± 0.2	7 ± 0.8 ^A^	7.55 ± 0.5	7.12 ± 0.4	7 ± 0.3 ^A^
2. SD	6.33 ± 0.1	6.52 ± 0.1	6 ± 0.0 ^B^	6.32 ± 0.4	6.30 ± 0.5	6 ± 0.5 ^B^	6.59 ± 0.1	6.77 ± 0.5	7 ± 0.3 ^A^
3. SD	6.22 ± 0.1	6.35 ± 0.2	6 ± 0.0 ^B^	6.22 ± 0.5	6.18 ± 0.4	6 ± 0.3 ^B^	6.35 ± 0.1	6.43 ± 0.4	6 ± 0.2 ^B^
4. SD	6.12 ± 0.0	6.18 ± 0.1	6 ± 0.2 ^B^	6.10 ± 0.3	6.14 ± 0.0	6 ± 0.2 ^B^	6.22 ± 0.3	6.30 ± 0.1	6 ± 0.0 ^B^
Average	7	7		6	6		6.67	6.65	
LSD (_%5_)	0.057 *								

Values with different letters indicate significant groups at the 5% level. SD: sowing date, LSD: least significant difference. *, **: significant at the 5% and 1% probability levels, respectively.

**Table 7:** Fatty acid content (%) of flax varieties according to four different sowing dates.

Sarı Dane					
	Palmitic Acid (C16:0)	Stearic Acid (C18:0)	Oleic Acid (C18:1)	Linoleic Acid (C18:2)	Alphalinolenic Acid (ALA, C18:3)
1. SD	6.36 **	6.30 **	20.37 **	13.25 *	53.00 **
2. SD	6.11 **	6.07 **	21.72	13.16 *	49.05 **
3. SD	5.82 *	6.00 **	20.62	12.23	48.34 *
4. SD	5.18	5.49 *	20.00	12.17	46.29
**Beyaz Gelin**					
	**Palmitic acid** **(C16:0)**	**Stearic acid** **(C18:0)**	**Oleic acid** **(C18:1)**	**Linoleic acid** **(C18:2)**	**Alphalinolenic acid** **(ALA, C18:3)**
1. SD	5.26 **	5.80 **	20.34 **	14.00 **	54.25 **
2. SD	5.78 **	6.35 **	20.45 *	13.48 *	52.74 **
3. SD	5.28	5.81 *	20.38 *	13.25 *	52.69 *
4. SD	5.18	5.34	20.15	12.98	51.58
**Kara Kız**					
	**Palmitic acid** **(C16:0)**	**Stearic acid** **(C18:0)**	**Oleic acid** **(C18:1)**	**Linoleic acid** **(C18:2)**	**Alphalinolenic acid** **(ALA, C18:3)**
1. SD	6.27 **	7.00 **	22.54 **	12.65 *	51.00
2. SD	5.78 *	6.75 **	21.72 *	11.75	49.35
3. SD	5.45	6.42	21.85	11.39	49.12
4. SD	5.40	6.35	20.98	11.24	49.03

SD: sowing date. *, **: significant at the 5% and 1% probability levels, respectively.

**Table 8:** Correlation analysis between traits measured at different sowing dates (2021 and 2022).

Agronomic Traits	Sowing Date	Plant Height	Number of Branches	Number of Capsule	Number of Seeds per Capsule	Capsule Length	Capsule Diameter	Oil Yield
Plant height	1. SD							
	2. SD							
	3. SD							
	4. SD							
Number of branches	1. SD	0.852 **						
	2. SD	0.781 **						
	3. SD	0.538 **						
	4. SD	0.545 **						
Number of capsule	1. SD	0.132	0.937 **					
	2. SD	0.156	0.846 **					
	3. SD	0.261	0.855 **					
	4. SD	0.178	0.731 **					
Number of seeds per capsule	1. SD	0.034	0.231	0.281				
	2. SD	0.019	0.145	0.158				
	3. SD	0.078	0.156	0.022				
	4. SD	0.036	0.078	0.015				
Capsule length	1. SD	0.025	0.032	0.218	0.035			
	2. SD	0.039	0.078	0.135	−0.062			
	3. SD	0.078	0.032	0.118	−0.055			
	4. SD	0.027	0.051	0.098	−0.042			
Capsule diameter	1. SD	0.182	0.036	0.035	0.087	0.528 *		
	2. SD	0.283	0.025	0.068	0.069	0.331		
	3. SD	0.016	0.019	0.032	0.038	0.282		
	4. SD	0.022	0.012	0.057	0.021	0.213		
Oil yield	1. SD	0.245	0.638 **	0.728 **	0.675 **	0.042	0.078	
	2. SD	0.149	0.527 *	0.615 **	0.593 **	0.035	0.055	
	3. SD	0.196	0.386	0.522 *	0.555 **	0.028	0.038	
	4. SD	0.285	0.218	0.486 *	0.439 *	0.015	0.020	

SD: sowing date, *,**: significant at the 5% and 1% levels, respectively.

## Data Availability

All data needed to conduct this study are provided within the manuscript.
